# Supplementary material for: Patterns of Intron Gain and Loss in Fungi
Source: PLoS Biol. 2004 Nov 30;2(12):e422. doi: 10.1371/journal.pbio.0020422 (PMC532390; doi:10.1371/journal.pbio.0020422)
Supplement: Table S1 — Also available at http://genes.mit.edu/NielsenEtAl/. (4.3 MB ZIP). [file pbio.0020422.st001.zip › NielsenEtAl/html/742.html]

AN5883.1.NCU07690.1.MG01728.1.FG09572.1


```
 CLUSTAL W (1.82) Multiple Sequence Alignments - Introns Inserted


Sequence 1: NCU07690.1	613 aa
Sequence 2: MG01728.1	631 aa
Sequence 3: FG09572.1	621 aa
Sequence 4: AN5883.1	627 aa
Alignment Length: 632 aa
Number Identitical Residues: 350 aa
Alignment Score (without introns) 17678


MG01728.1 	MHIRDMLAETEKTGKPSFSFEYFPPKTAQGVQNLYDRMERMYHFGPKFIDITWGAGGRIA
NCU07690.1	MHIREMLADAERTKAPSFSFEYFPPKTAQGVQNLYDRMDRMYNFGPKFIDITWGAGGRIA
FG09572.1 	MHIKDMLNDAERSGQPSFSFEYFPPKTAQGVQNLYDRMDRMYNLGPKFIDITWGAGGRIA
AN5883.1  	MHIKEKLAQNEAAGKIGISFEFFPPKTAQGVQNLYDRMDRMHGLGPSFIDITWGAGGRLS
          	***:: * : * :   .:***:****************:**: :**.***********::

MG01728.1 	ELTCEMVTQAQTYFGLETCMHLTCTDMGVEKVNDALSKAYKAGCTNILALRGDPPRDKEK
NCU07690.1	ELTCEMVAQAQAYLGLETCMHLTCTDMGEEKVNNALQRAYKAGCTNILALRGDPPREKER
FG09572.1 	ELTCEMVLQAQAVYGLETCMHLTCTDMGVEKVNDALLKAYKAGCTNILALRGDPPRAQDK
AN5883.1  	DLTCEMVNVAQSVYGLETCMHLTCTDMPQERVDAALQAAYKAGCTNILALRGDPPREKEV
          	:******  **:  *************  *:*: **  ****************** :: 

MG01728.1 	WEAAQDGFNYAKDLVSHIRKTYGDHFDIGVAGYPEGCDDNKDEDLLLDHLKEKVDMGASF
NCU07690.1	WEAAEGGFQYARDLVAHIRKLYGDHFDIGVAGYPEGCDDNKDEESLLDHLKEKVDMGASF
FG09572.1 	WEAADGGFQYARDLVKHIRNTYGNHFDIGVAGYPEGSDDNKNEDELLDHLKEKVDMGATF
AN5883.1  	WEAADGGFRYAKDLVKYIREKYGNHFCIGVGGYPEGADDNSDVDQLIDHLKEKVDAGSSF
          	****:.**.**:*** :**: **:** ***.*****.***.: : *:******** *::*

MG01728.1 	IVTQMFYDADNFVRWVGRVRERGITVPIIPGIMPIATYASFLRRANHMQARIPEEWLQRL
NCU07690.1	IVTQMFYDVDNFLRWVKRVRERGITIPIIPGIMPIATYASFIRRANHMNCKIPEEWMAKL
FG09572.1 	IVTQMFYDADNFIRWVKRVRERGITIPILPGIMPIATYASFLRRANHMQAKIPQEWLDAL
AN5883.1  	IVTQMFYDTDNFIKWVEKCRAKGINVPIIPGIMPIQTYAAFIRRANWTKTHIPPDWLEAL
          	********.***::** : * :**.:**:****** ***:*:****  : :** :*:  *

MG01728.1 	EPIKTDDAAVRIVGRQLVVELCRKILAAGIHHLHF~YTMNLAQSTALILEDLDWLPSPNK
NCU07690.1	EPVKNDDVAVREIGKTLVAEMCRKIVDAGIRHLHF~YTMNLAQATRMVLEELDWLPSPNR
FG09572.1 	EPVKNDDVAVRNIGKTLVANMCRKLLANGIHHLHF~YTMNLAQATRMLLEELQWAPSADR
AN5883.1  	EPVKNDDAAVKQVGKRLIADMCRRLIASGIKHLHF2YTMNLAQSTQLVLEELELTPTAEA
          	**:*.**.**: :*: *:.::**:::  **:**** *******:* ::**:*:  *:.: 

MG01728.1 	PLKHALPWKQSLGLGRREEDVRPIFWRNRNKSYVMRTQDWDEFPNGRWGDSRSPAFGELD
NCU07690.1	PLKRALPWKQSLGSGRRDEDVRPIFWRNRNKSYITRTQDWDEFPNGRWGDSRSPAFGELD
FG09572.1 	PLQHALPWKQSKGLGRREEDVRPIFWRNRNKSYVIRTQDWDEFPNGRWGDSRSPAFGELD
AN5883.1  	PIPRPLPWRPSLGLNRREEDVRPIFWRNRNSSYIARTAIWDEFPNGRWTDSRSPAFGELE
          	*: :.***: * * .**:************.**: **  ********* **********:

MG01728.1 	AYGIGLLGTNEQNRKKFGEPKSVKDIATLFVRYVQKEVDTLPWSESPLDAEAEQIRDDLI
NCU07690.1	AYGIGLTGSNELNRKKWGEPQTVKDIANLFVRYLNQELEYLPWSEAPISSEAELIKDDLI
FG09572.1 	AYGIGLTGSNEANRKKWGEPKAIQDIAELFVSYLNNKIESLPWSESPLTTEADEIRDDLI
AN5883.1  	LYGVGLKGTNEQNIKLWGEPNSLRDLAEVFVRFLEGNLDRLPWSDSPITPETSAIKDALV
          	 **:** *:** * * :***::::*:* :** ::: ::: ****::*: .*:. *:* *:

MG01728.1 	DLNLRGLITINSQPAVNGVRSTHPIHGWGPPNGYVYQKAYLELLVHPAIFEQLKERIHDH
NCU07690.1	ALNNRGLITINSQPAVNGVKSTHPVHGWGPANGYVYQKAYLELLVSPELFPEIKRRIEDH
FG09572.1 	ELNKRGLLTINSQPAVNGVKSSHPIHGWGPDNGYVYQKSYLELLVHPGVFDKMISRIENH
AN5883.1  	ELNRRGFFTINSQPAVNGVKSSHPVFGWGPKNGFVYQKAYLELLVPPYVIDELIARIEAN
          	 ** **::***********:*:**:.**** **:****:****** * :: ::  **. :

MG01728.1 	PDLTYYAVTKSGNLHTNATYEGPNAVTWGVFPGKEIVQPTVVENISFLAWKDEAFQLGME
NCU07690.1	PDITYYAVTKSGNLHTNAPSEGPNAVTWGVFPGKEIVQPTIVESISFLAWKDEAFQLGLE
FG09572.1 	PDLTYYAVTKDGELRSNVTSDGPNAVTWGVFPGKEIVQPTIVESISFLAWKDEAFRLGVD
AN5883.1  	EDLTYHAVTKKGELRTNTR-DSPNALTWGIFAGREVVQPTIVETVSFLAWKDEAYQLGED
          	 *:**:****.*:*::*.  :.***:***:*.*:*:****:**.:*********::** :

MG01728.1 	WARCHDQNTPSRILIQSMMQEWYLVNIV1NNDFHAPRTIFDTLKGLTVPDIDTEIVPPAL
NCU07690.1	WARCYDADTPSRLLIEEIMDKWYLINIV1NNDFHQPRTIFEILNDLSLPDLEKVYTP---
FG09572.1 	WAHCYDISSPSRALLEGVMNDWYLVNIV1NNDFHDNKTIFELLKGLEVNDLTTPATPLS-
AN5883.1  	WAKCHEASSPTRKLLQGIMDSWYLVNIV1NNDFHNTYDLFELFKDLNVKDLELEISGGIT
          	**:*:: .:*:* *:: :*:.***:*** *****    :*: ::.* : *:         

MG01728.1 	PTAVPEAAAINGERNGERNGAQANGATAAVTAS-
NCU07690.1	-----------AHTNGATNGVPEASTVPQ-----
FG09572.1 	----------EPVANGAVDTEPIANGTAVTAAAN
AN5883.1  	----EDKAHHNGTANGATNGTTSNGTAAEQPVKN
          	     . :  .   **  :     . ..  .  .
```
